# 3D genome organization and epigenetic regulation in autoimmune diseases

**DOI:** 10.3389/fimmu.2023.1196123

**Published:** 2023-06-06

**Authors:** Yueqi Qiu, Delong Feng, Wenjuan Jiang, Tingting Zhang, Qianjin Lu, Ming Zhao

**Affiliations:** ^1^ Institute of Dermatology, Chinese Academy of Medical Sciences and Peking Union Medical College, Nanjing, China; ^2^ Key Laboratory of Basic and Translational Research on Immune-Mediated Skin Diseases, Institute of Dermatology, Chinese Academy of Medical Sciences, Nanjing, China; ^3^ Department of Dermatology, The Second Xiangya Hospital of Central South University, Changsha, China; ^4^ State Key Laboratory of Natural Medicines, School of Basic Medicine and Clinical Pharmacy, China Pharmaceutical University, Nanjing, China

**Keywords:** 3D genome, autoimmune disease, GWAS, gene expression, SNP, epigenetic

## Abstract

Three-dimensional (3D) genomics is an emerging field of research that investigates the relationship between gene regulatory function and the spatial structure of chromatin. Chromatin folding can be studied using chromosome conformation capture (3C) technology and 3C-based derivative sequencing technologies, including chromosome conformation capture-on-chip (4C), chromosome conformation capture carbon copy (5C), and high-throughput chromosome conformation capture (Hi-C), which allow scientists to capture 3D conformations from a single site to the entire genome. A comprehensive analysis of the relationships between various regulatory components and gene function also requires the integration of multi-omics data such as genomics, transcriptomics, and epigenomics. 3D genome folding is involved in immune cell differentiation, activation, and dysfunction and participates in a wide range of diseases, including autoimmune diseases. We describe hierarchical 3D chromatin organization in this review and conclude with characteristics of C-techniques and multi-omics applications of the 3D genome. In addition, we describe the relationship between 3D genome structure and the differentiation and maturation of immune cells and address how changes in chromosome folding contribute to autoimmune diseases.

## Introduction

Among the most important mechanisms for regulating cellular life is DNA, the genetic material inside the cell. The interaction of DNA and histone octamers in the form of ribosomes results in the development of chromatin fibers. Scientists have thoroughly aligned, interpreted, and annotated the characteristics and functions of various sequences in the genome, including coding sequences, non-coding sequences, transcripts, and non-coding RNAs after the completion of the Human Genome Project (HGP) (1) and the encyclopedia of DNA elements (ENCODE) (2). Gene bodies, promoters, and some important *cis*-regulatory DNA elements including enhancers, silencers, and insulators are also found in linear chromatin (2). As we know, the chromatin fibers are compressed into the nucleus in an orderly three-dimensional (3D) structure at various levels. The eukaryotic genome has a hierarchical organization instead of being arranged linearly, which allows the transcriptional and regulatory machinery to reside in specific locations, facilitating their efficient function (3). In 2002, Dekker proposed the chromosome conformation capture (3C) technology (4) to study the geographic organization of the genome inside the cell nucleus. In the following years, 3C-based derivative sequencing technologies were rapidly developed, such as chromosome conformation capture-on-chip (4C) (5), chromosome conformation capture carbon copy (5C) (6), high-throughput chromosome conformation capture (Hi-C) (7), and chromatin interaction analysis by paired-end tag sequencing (ChIA-PET) (8). The effects of DNA folding resulting in physical interactions could have a significant impact on gene regulation, allowing regulatory elements to interact with gene promoters mediated by specific chromatin structure-associated proteins like CCCTC-binding factor (CTCF) and cohesin. Furthermore, recent discoveries suggest that 3D chromatin structure is involved in a variety of cell types and biological activities, including embryonic development, immunity, and disease susceptibility (9). For example, the 3D chromatin structure of the fertilized egg is gradually built up during the two-cell stage and, by the time of the inner cell mass, is basically similar to that of embryonic stem cells (10). Chen et al. (11) investigated 8,928 samples from 33 tumor types and discovered that enhancers were activated in the majority of cases, which were generated by particular topologically associating domains (TADs), where regulators that are linearly distant may fold into 3D structures through chromatin to reach spatial closeness, leading to cancer development.

The study of the 3D genome focuses on the fundamental principles of chromatin folding, assembly, and regulation in the nucleus as well as the mechanisms that govern genome regulation on a genome-wide scale. In early genomics research, genome-wide association studies (GWASs) have made significant advancements in identifying variations associated with complicated genetic disorders, including autoimmune diseases (12). Single-nucleotide polymorphism (SNP) and chromatin rearrangements involving deletions, duplications, insertions, inversions, and translocations could potentially interfere with gene expression and protein synthesis. Large-scale GWASs have shown that 80%–90% of variants are in non-coding regions (13). Various genetic variations are believed to affect disease risk by influencing gene expression, which can differ depending on the cell type and cellular state (14). In addition, approximately 60% of the causal variants associated with autoimmune diseases have been mapped to enhancer regions in immune cells (15). The process of DNA looping is responsible for transporting transcription factors (TFs) to gene promoters, which are often positioned on the same chromosome, but at different distances. An important advantage of 3D genomics is the ability to link diverse loci across the genome, which is vital for linking GWAS-identified expression quantitative trait loci (eQTLs) with potential target areas. Several studies have demonstrated that disease-associated SNPs may serve as marker to uncover regulating elements that play a significant role in disease susceptibility (16–20). For example, a risk variant for systemic lupus erythematosus (SLE), rs2280381, is found in an area of the distal enhancer that regulated *IRF8* expression by spatially interacting with the *IRF8* promoter and influencing methylation levels. DNA methylation, histone post-translational modification, and non-coding RNAs have been extensively explored at the epigenetic level in many autoimmune diseases (21, 22). Despite significant progress in understanding the pathogenesis of these diseases, there have been relatively few 3D genomic studies conducted in autoimmune diseases compared to psychiatric disorders and cancer.

This review summarized current concepts in 3D genome organization and multi-omics research techniques and applications of the 3D genome. We focus on how the 3D organization of genomes has an impact on the feature of immune cells of innate and adaptive immunity. Furthermore, we discuss how variants in non-coding regions, as well as epigenetic changes, might disrupt spatial genomic folding and generate an abnormal state of autoimmune diseases, as well as the functional consequences of autoimmune disease­associated variants and chromosomal rearrangements. Furthermore, following changes in gene expression and cellular function regulation throughout disease progression may give clues for developing new biomarkers and designing focused therapy regions.

## Hierarchical view of 3D genome organization

**Figure 1:**
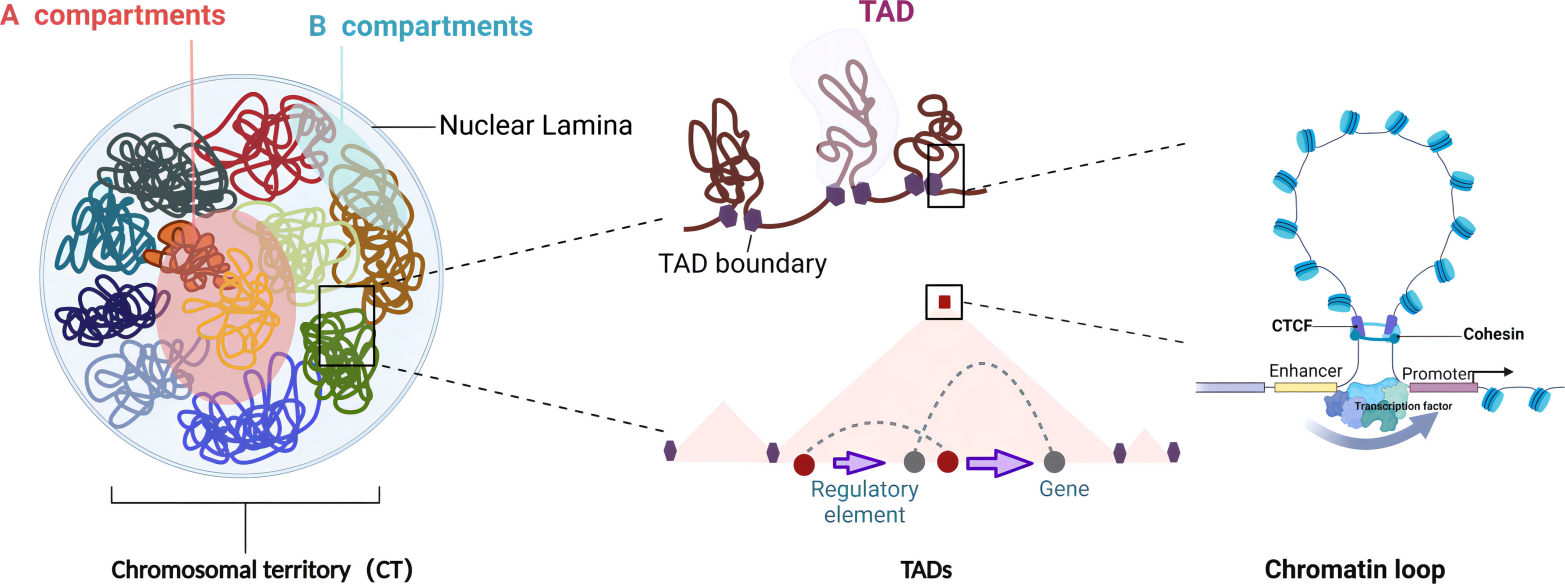
Features of 3D genome organization. The 3D genome hierarchical units of chromatin in the nucleus are in descending order: chromosomal territories (CTs) are the universal domain of the genome, with each chromosome occupying a separate and non-overlapping area in the nucleus. Chromatin compartments are composed of two compartments of A and B with different appearances in the spatial structure of the genome. The A compartments are open chromatin compartments with mostly euchromatin, showing high concentration and expression of genes. The B compartments are closed chromatin compartments of repressed transcription regions with mostly heterochromatin. Topologically associated domains (TADs) are stable spatial structural units in the nucleus, which act as functional units of genome folding and guide the regulation of long-range regulation. Chromatin loops are usually formed by distant interactions between promoters and distal enhancers or regulatory elements, which are the most elaborate structural and functional units directly regulating gene expression (created with BioRender.com).

Chromosomes are not randomly distributed within the nucleus but tend to occupy distinct, non-overlapping regions known as chromosome territory (CT), representing the highest level of chromatin organization (23). Chromosomes with high gene density are found more centrally in the nucleus, whereas chromosomes of low gene density are predominantly located toward the nuclear periphery (24, 25). The CT organization is critical for nuclear functions, including transcription, replication, RNA processing, and DNA damage repair (25). Mehta et al. showed that DNA damage caused a large-scale spatial repositioning of CT regions, including a partial transfer of CTs from the interior to the exterior of the nucleus. Interestingly, this is a reversible process in which chromosome territories reoccupy positions similar to those in intact control cells after repair, indicating the stability of CTs in the nucleus (26). Chromatin is separated into two multi-megabase (Mb) compartments at the sub-chromosomal level. The A compartments are distinguished by open chromatin areas that are rich in active histone marks and genes with high levels of transcription and are found internally in the nucleus. The B compartments are often located at the nuclear periphery and are associated with low levels of gene expression and histone modifications related to active chromatin, such as acetylation. As a result, the B compartments are transcriptionally repressed (27). Although chromatin compartments are generally permanent in the nucleus, there is significant interconversion between the A and B compartments throughout growth and disease development, suggesting that chromatin compartments are highly malleable and correspond with the expression of cell-specific genes. On a smaller scale, chromosomes are organized into TADs, the sub-megabase regions with a high frequency of self-interactions (28, 29). Normally, TADs are separated from surrounding domains by boundary regions (also known as borders) where local interactions are reduced. TAD borders are enriched in housekeeping genes, transfer RNA, the CTCF–cohesin complex, transcription start site (TSS), and active transcriptional indicators like H3K4me3, H3K36me3, and H3K9me2 (9, 30). Cell-specific TAD boundaries divide adjacent domains and restrict the interactions between promoters and enhancers. After the genetic deletion of a boundary, nearby TADs tend to fuse, resulting in abnormal connections between regulators and genes (31). In some cases, shifting genes to a new TAD will result in ectopic interactions with regulatory elements, leading to an alteration of their expression profile. Additionally, nearly all heterochromatin is found close to the nucleus and nucleolar peripheries, which are known as lamina-associated domains (LADs) and nucleolus-associated domains (NADs), respectively (32). LADs make up approximately 40% of the genome and mainly consist of gene-depleted regions of transcriptionally silenced chromatin enriched in histone H3K27me3 (33). NADs have higher H3K9me2 and lower H3K27me3 contents than regions that only interact with the nuclear lamina (34). Chromatin loops, which are frequently linked promoters and enhancers, are more elaborate structural units than TADs. Gene promoters and enhancers can be either looped together to upregulate gene expression or excluded from loops to initiate borders and suppress gene expression (35). The presence of CTCF and the cohesin subunits RAD21 and SMC3 in more than 86% of the loops suggests that CTCF and cohesin are involved in loop anchor formation (27). In the loop extrusion model, the structural maintenance of the chromosome, like the cohesin complex, pushes the linear DNA outward until the complex comes into contact with the convergent CTCF bound to loop anchor sequences (36, 37). Moreover, certain TFs like SATB1, P300, YY1, EKLF, OCT4, and SOX2 have been reported to play critical roles in chromatin looping control (38–43). The 3D genome structure controlling distal connections allows complex gene regulation networks to form, enabling multiple enhancers to interact with one promoter or a single enhancer to contact numerous promoters (44) (**Figure 1**).

## Multi-omics research techniques and applications in 3D genome

Imaging-based approaches were used in the majority of early 3D genomics studies (45), including microscopy and fluorescence *in situ* hybridization (FISH), which makes it possible to visualize the spatial length between two or more loci by hybridizing fluorescently labeled probes to DNA after fixation and moderate denaturation (46, 47). These tools may directly quantify the spatial separations between genomic loci in individual cells, but the throughput, resolution, and genome coverage are limited. Recently, super-resolution and cryogenic electron microscopy allowed us to observe 11-Å structures for 30-nm chromatin fibers (48). Other approaches based on C-technologies have been implemented to specifically measure the physical connection between DNA segments (4). Chromatin is first cross-linked to maintain interactions between the proximal areas of DNA and binding proteins. Subsequently, the cross-linked chromatin is disassembled into DNA fragments by restriction enzymes. The remaining DNA regions that are in close spatial proximity can be enzymatically ligated together to form a chimera DNA strand, a procedure known as proximity ligation. Following de-crosslinking, chimeric DNA is suitable for downstream analysis of 3D chromatin with PCR, DNA microarrays, or DNA sequencing (13). The initial version of the C-technology, known as 3C, could verify interactions between only two pre-determined genomic loci and identify known DNA interactions using fluorescence quantitative PCR (49). 4C technology can determine the interactions between specific genomic baits and several fragments in the form of a ring with only a pair of primers (50). 5C uses a highly multiplexed ligation-mediated amplification that allows the amplification of 3C ligation compounds. Either microarray or high-throughput DNA sequencing is used to examine the resulting 5C library of ligated primers (51). As the many-to-many technique, 5C has been applied to 1% of the human genome to explore long-range interactions between DNA sequences and distal regulators (52). High-resolution chromatin interaction profiles can be produced by Hi-C, which combines biotin enrichment with high-throughput sequencing to examine genome-wide intra- or interchromatin interactions at specific geographical regions (7). The resolution of Hi-C is constrained by sequencing depth. For example, 8.4 million, 2 billion, and 6.5 billion total reads would be needed to attain 1-Mb, 10-kb, and 1-kb resolution, respectively (27, 49). A Hi-C-based method, Micro-C, enables mononucleosome resolution chromosome folding maps using micrococcal nuclease (MNase) rather than restriction enzymes (53). Further, Micro-C XL increases the signal-to-noise ratio by isolating insoluble chromatin and using long cross-linkers (54). Like several high-throughput sequencing methods, different derivative technologies of Hi-C such as DNase Hi-C, *in situ* Hi-C, and BL-Hi-C have been improved in order to increase the resolution or effectiveness of proximity ligation and also to reduce the bias caused by the use of restriction enzymes (27, 55, 56).

Furthermore, technologies based on combining 3C and Hi-C with oligonucleotide capture, single-cell isolation, and high-throughput sequencing have been developed, which have greatly facilitated the study of the chromatin conformation in the regulation of spatial and temporal specific expression of genes. In the Capture-C or Capture Hi-C (CHi-C) technique, a 3C or Hi-C library is subjected to capture by hybridization with pools of DNA or RNA oligos to concentrate ligation products that fit specific sites (57, 58). With less library complexity, these techniques enable a significant rise in the number of distinguishable interactions within specific areas without ultra-deep sequencing. Chromatin immunoprecipitation-loop (ChIP-loop), a method for studying protein-mediated chromatin interactions, combines ChIP and 3C techniques (59). ChIA-PET is a scale-up of the ChIP-loop technique that identifies genome-wide chromatin connections without preference and is sequenced after immunoprecipitation of DNA–protein complexes with antibodies specific to the target protein (60). *In situ* Hi-C followed by chromatin immunoprecipitation (HiChIP) is a novel technique that constructs a sequencing library by Tn5 transposase with reduced starting material (**Table 1**).

**Table 1:** The characteristics of multi-omics research techniques and applications in 3D genome chromatin structures.

Type	Assay	Features and application	References
C technologies	3C	Measuring long-range interactions between two targets by quantitative PCR	(49)
	4C	Identifying several DNA regions interacting with a target gene by microarray	(50)
	5C	Multiplexed conformation capture by amplifying DNA chimeric fragments with T3 and T7 adaptors followed by deep sequencing	(51)
	Hi-C	Detecting all interactions and spatial organization of DNA by deep sequencing after purification by streptavidin beads	(7)
	Micro-C/Micro-C XL	Investigation of chromosome folding on length scales from the nucleosome to the entire genome	(53, 54)
	Capture C	Capturing chromatin interaction among target DNA sites based on oligonucleotide probe	(58)
	Capture Hi-C	Measuring whole genome interacting regions with target DNA fragments by target sequence in Hi-C library based on oligonucleotide probes with lower background signal	(57)
	ChIA-PET	Measuring specific protein-mediated whole interactions by combining with ChIP and Hi-C	(60)
	HiChIP	Combined with ChIP, Hi-C, and Tn5; reduction in input required for construction of a sequencing library by Tn5	(61)
Chromatin accessibility	DNase-seq	Digested by DNase I; data on chromatin accessibility were sequenced by high- throughput sequencing	(62)
	FAIRE-seq	Fragmentation by ultrasonic resulting in sequence cutting without heterosexuality; information on chromatin accessibility was obtained by high- throughput sequencing	(63)
	MNase-seq	Digested by MNase; data of chromatin accessibility were sequenced by high- throughput sequencing	(64)
	ATAC-seq	Digested by Tn5; information on chromatin accessibility was obtained by high- throughput sequencing	(65)
	scATAC-seq	Application of ATAC-seq technology at the single- cell level	(66)
Whole genome	GWAS	Discovering genetic variation influencing the trait	(67)
Transcription	RNA-seq	Whole-transcriptome RNA sequencing	(68)
	GRO-seq	Identifying transcription of enhancer RNA	(69)
	QTLs	Relationship between SNPs and the level of gene expression	(70)
Histone marks and Protein binding	ChIP-seq	Ultrasonic fragmentation; immunoprecipitation with particular antibodies to identify histone modifications and transcription factors, followed by next-generation sequencing	(71)
	CUT&Tag	Digested by Tn5; detection of histone modifications or DNA-bound regulatory proteins by immunoprecipitation with specific antibodies, followed by next-generation sequencing	(72)

3C, chromosome conformation capture; 4C, chromosome conformation capture-on-chip; 5C, chromosome conformation capture carbon copy; Hi-C, high-throughput chromosome conformation capture; ChIA-PET, chromatin interaction analysis by paired-end tag sequencing; HiChIP, in situ Hi-C followed by chromatin immunoprecipitation; ChIP, chromatin immunoprecipitation; ATAC-seq, assay for transposase­accessible chromatin using sequencing; GWAS, genome-wide association study; QTLs, quantitative trait loci; ChIP-seq, chromatin immunoprecipitation followed by sequencing; CUT&Tag, cleavage under targets and tagmentation.

The degree to which *cis*-regulatory elements and *trans*-acting factors can physically interact with chromatinized DNA is referred to as chromatin accessibility (73). Furthermore, post-translational chemical modifications of chromatin are dynamically changing across developmental stages and cell types and are associated with chromatin accessibility, indicating the function of specific genomic regions (74). Deoxyribonuclease I (DNase I) hypersensitive site sequencing (DNase­seq) is a method employing high-throughput sequencing to interrogate DNase hypersensitive sites to detect potentially active chromatin areas, while prior knowledge of histone modifications and TSS are not required (75). FAIRE (formaldehyde-assisted isolation of regulatory elements) combined with high-throughput sequencing (FAIRE-seq) is similar to DNase-seq, whereas DNA fragmentation is performed by sonication instead of ribonuclease and then purified by phenol/chloroform (63). Although the operations of FAIRE-seq are reasonably simple and convenient, they are not favorable to detect sensitive open chromatin areas because of unmanageable ultrasonic mechanical damage and unpredictable accuracy of cleaving. MNase-seq cleaves and eliminates accessible DNA not protected by proteins by using the endo-/exo-nuclease. MNase, a small-molecular-weight enzyme, is widely used to isolate fragments spanning single nucleosomes that are inaccessible to DNase I or Tn5 with a high efficiency of cleaving internucleosomal DNA (64). MNase hypersensitivity sequencing (MH-seq) provides a further improvement in the resolution of open chromatin mapping. Compared to DNase I and Tn5, some specific genomic region- enriched MH-seq reads show unique epigenetic marks H3K27me3 and DNA methylation (76). In addition, an assay for transposase­accessible chromatin using sequencing (ATAC-seq) introduces sequencing adaptors into open chromatin areas using a hyperactive Tn5 transposase (65). Compared with DNase­seq, FAIRE-Seq, and MNase-Seq, cell requirements for ATAC-seq libraries are reduced from 10^6^ ~10^7^ to 5,000~50,000 (77). Advancements in single-cell sequencing technology have led to the development of various methods for assessing chromatin accessibility in individual cells, which are based on library preparation procedures used in ATAC-seq and DNase-seq. Zhang et al. studied 30 types of adult human tissue using scATAC-seq and identified 111 different cell types based on similarities in chromatin (78). Combining their datasets with single-cell chromatin accessibility data from 15 types of fetal tissue (79), 1.2 million *cis*-regulatory elements covering 14.8% of the genome for 222 different cell types have been determined, providing a comprehensive overview of gene regulatory programs in human cells in different tissues, life stages, and organ systems. Moreover, a variety of high-resolution chromatin accessibility assays based on 3C or Hi-C, such as HiCAR (Hi-C on accessible regulatory DNA) (80), DNase Hi-C (81), HiCoP (82), and Ocean-C (83), have been developed to profile accessible chromatin regions and to reveal the interaction between *cis*-regulatory elements.

**Figure 2:**
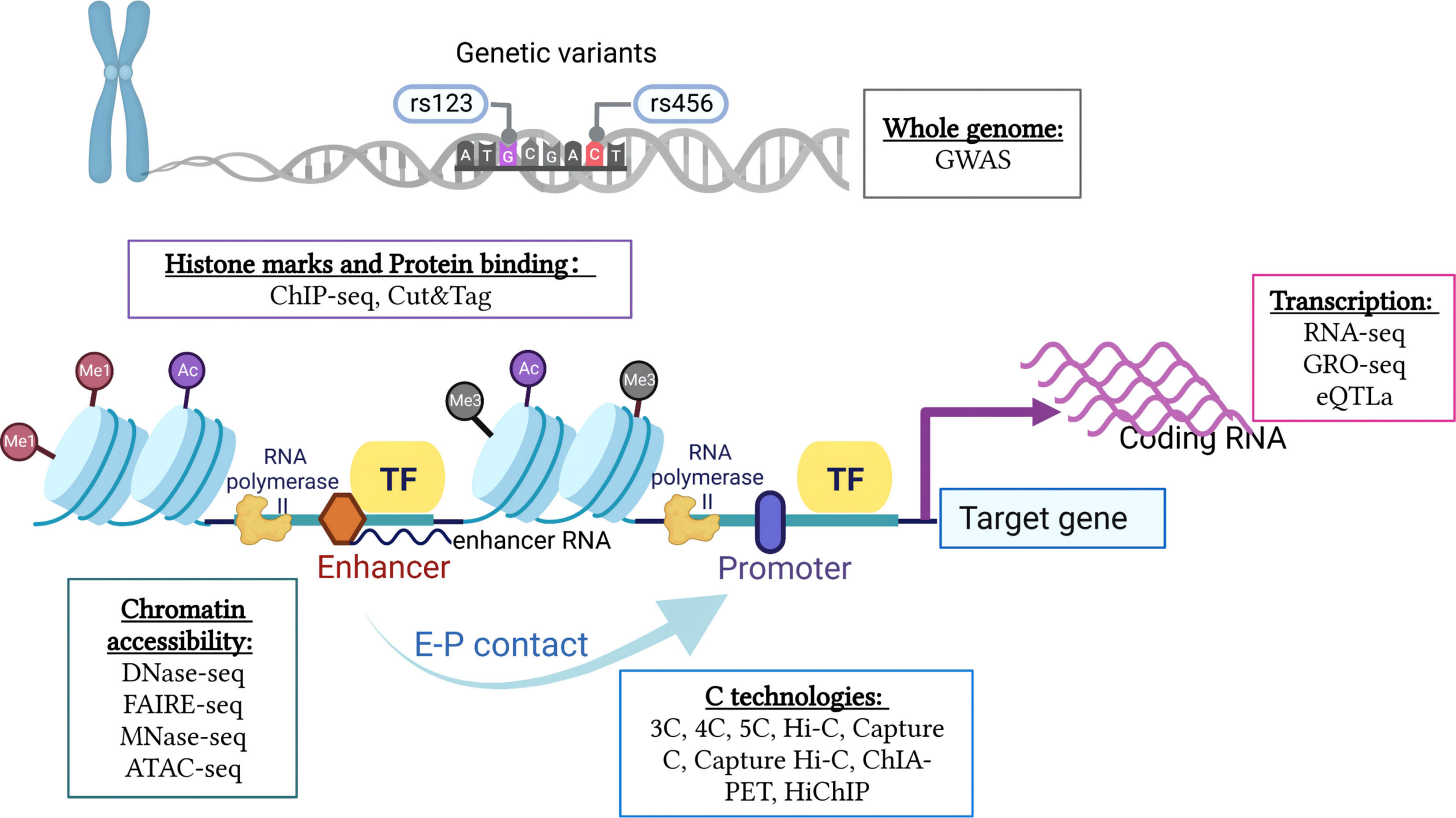
Integration of multi-omics research techniques of 3D chromatin and genome organization. A comprehensive analysis of the interrelationships between different regulatory components and gene function requires integration of multi-omics data, including genetic variation of the whole genome, chromatin accessibility, histone modifications and protein binding, gene transcription levels, and chromatin conformation (created with BioRender.com).

To accurately assess the interrelationships between different regulatory components and gene function during cell development and differentiation, the integration of multi-omics data to define the biological effects of associated genes, transcriptional regulatory elements, and other biomolecules involved in the transcriptional regulation of specific genes is required. Numerous loci have been identified as statistically associated with various diseases and traits based on GWASs. However, because more than 80% of disease-associated variants are found in non-coding areas, it is difficult to ascertain the biological mechanism behind a genetic risk factor (14). Generally, QTLs indicate genes that are involved in quantitative traits (97). Several QTL analyses have been conducted to assess the polygenic nature of phenotypes and intermediate phenotypes, like gene expression. Recent advances in next-generation sequencing techniques have enabled the analysis of histone modifications and chromatin structures. For instance, RNA-seq reveals the level of gene expression. Chromatin immunoprecipitation followed by sequencing (ChIP-seq) and cleavage under targets and tagmentation (CUT&Tag) identifies distinct nucleosome properties or TSS. GWAS variants in non-coding or intergenic genomic regions can be linked more precisely to the genes they affect (98) (**Figure 2**).

## Insights into lymphocyte development and immune response from dynamic 3D genome

Different types of cells make up the immune system with specialized functions, concluding rapidly responding innate immune cells and highly specialized lymphocytes (99). It is noteworthy that impaired transcriptional control in lymphocytes could have a detrimental effect on immune cell development, which has been observed in a variety of diseases such as cancers, immunodeficiencies, and autoimmune disorders (100). In addition, 3D genome folding is involved in the regulation of gene expression, immune cell differentiation, activation, and dysfunction (101).

B-cell receptors (BCRs), consisting of pairs of immunoglobulin heavy (IgH) and immunoglobulin light (IgL) chains, and T-cell receptors (TCRs), composed of either an α and β chain or a γ and δ chain, confer the ability to detect foreign antigens (102). The antigen receptor segments composed of variable (V), diversity (D), and joining (J) gene segments are recombined during B- and T- cell development, in order to create the antigen-specific variable domains of the Ig/TCR chains (103). Chromosome openness reorganizes locally at recombination signal sequences (RSSs) and globally across antigen receptor loci with V (D)J recombination in a time-dependent manner. V (D)J recombination is initiated by the protein complex derived from the recombination activating gene 1 (RAG1) and RAG2 in recombination centers, where gene segments become accessible to the complex. DNA is recombined RSSs adjacent to segments of V, D, and J genes by RAG recombinase (104). Given that diverse V segments are scattered across 0.6–3 Mb of linear genomic space at IgH and TCR sites, rearrangements of these segments can be difficult. 3C-based methods have shown that the looping of DNA sequences underlies cell compaction and enhances interactions between V and (D)J segments (105). CTCF binding sites in the V region are forward- oriented, while reverse-oriented CTCF binding sites flank the recombination center (37). Through the cohesin-mediated loop extrusion constrained by convergent CTCF sites, diffusion-based synapsis of accessible V RSSs by D/J RSSs bound to RAG would be enhanced in the compacted structure (37, 106).

Significant alterations of genomic topology accompany early B- cell development (107). It has been shown that a transcriptionally inactive gene that encodes early B-cell TF (*Ebf1*) is sequestered in the nuclear lamina of progenitor cells switching between an inactive B compartment and an active A compartment. By developing new intradomain and interdomain interactions at the pro-B- cell stage, B-lineage transcriptional signatures are developed (108). In addition to regulating gene expression and chromatin status, TFs appear to reconfigure the 3D genome in compartments of early B cells. TF binding sites’ co-localization forms two interaction hubs within nuclear space. First, a large portion of the cell-type-invariant structural loops in the genome is produced by short-range contacts between CTCF and the cohesin complex. Second, long-range interactions between TFs such as E2A and PU.1 with enhancer-binding histone acetyltransferases P300 acted within and between chromatin domains (108). Paired box 5 (PAX5), an example of a TF that specifies a particular lineage, is crucial for establishing the B- cell lineage by triggering B cell-specific genes and suppressing other immune cell genes (109). PAX5 binding sites were supposed to regulate global 3D genomic organization because only 47% of these sites were located in genes or promoters (107). A total of 7,810 differential chromatin interactions were identified between the wild-type (WT) and *Pax5*−/− pro-B cells, while 83% of differential interactions are weakened or abolished in the absence of *Pax5* (107). In addition, PAX5 enhances cohesin’s residence time on chromatin and facilitates extended loop extrusion by suppressing the expression of WAPL, specifically in pro-B and pre-B cells (110).

The thymus serves as the primary site for T- cell development, where the double-negative stage of growth takes place, during which neither CD4 nor CD8 is expressed. T cells follow a stepwise differentiation pathway monitored by Notch signals and combinations of TFs (such as BCL11B and RUNX), resulting in naïve CD4^+^ and CD8^+^ T cells (111). BCL11B is crucial for silencing alternative lineage programs in double-negative thymocytes, as revealed by BCL11B binding to regions with repressive H3K27me3 enrichment. Substantial intra-TAD interaction was also mediated by BCL11B as cells differentiate toward the double-positive stage (112). Similar to *Ebf1* in B- cell development, the *Bcl11b* locus is repositioned from the B compartment to the A compartment during T-cell differentiation, which is mediated through transcription of a non-coding RNA known as thymocyte differentiation factor (*ThymoD*). *ThymoD* transcription stimulates CpG residue demethylation and enhances the recruitment of cohesin, bringing the *Bcl11b* promoter and enhancer together into a loop domain (113). Recently, another critical TF, T- cell factor 1 (TCF1), has been implicated to weaken or disrupt the TAD border insulation through recruiting NIPBL and cohesin complex at active enhancers. This mechanism improves the interplay of target genes and regulatory elements on insulated domains in T- cell progenitors (114). Taken together, the aforementioned investigations demonstrate the critical role of chromatin-associated protein binding, transcription factor-mediated transcriptional regulation, and 3D chromatin rearrangement in lymphocyte development.

Inflammatory response genes are rapidly upregulated when receptors recognize specific molecules associated with pathogens, which are associated with a network of enhancers established by lineage-determining transcription factors (115). While there is little change in TADs and compartments in response to innate immune triggers, enhancer–promoter contacts in the 3D genome are essential for the rapid activation of target genes upon stimulation (101, 116, 117). Unlike innate immune cells, naïve T cells and B cells need more time to establish an inflammatory gene expression program after accepting antigen recognition and co-stimulatory signals (101). In naïve T cells, TCF1 and CTCF modulate the 3D genomic architecture to control a similar set of genes (118). Dynamic 3D chromatin interactions are thought to be involved in the activation of T and B cells and the initiation of adaptive immune response in several studies. TAD architecture of human T cells remains generally constant for initial periods, but 72 h after activation, TAD partition displayed smaller chromatin domains than those in resting cells (119). CD4^+^ T cells are at the center of the adaptive immune system, which is phenotypically specialized in various ways by cytokine stimulation, for example, Th1, Th2, and Th17 cells. T-bet TF regulates the differentiation of naïve CD4^+^ T helper lymphocytes by increasing transcription of Th1-specific genes (such as *IFN-γ*) and suppressing transcription of Th2-specific genes (such as *IL-4*) (120). 3C experiments demonstrate that T-bet is necessary for the direct development of chromatin loops or tethers at the *IFN-γ* gene locus (121). Th1 and Th2 cell subset-specific interactions of *Ifng* and *Il4* genes appear to be regulated by stimulation-induced signal transducer and activator of transcription (STAT) TFs across the genome. *STAT4*-deficient cells are unable to produce Th1-selective *Ifng* contact due to the prevented 3D genome reorganization (122). The basic leucine zipper TF activating transcription factor-like (BATF), a necessary TF for the generation of Th17 and follicular helper T cell (TFH) cells, recruits CTCF to lineage-specifying gene loci to form a chromatin looping in the transcriptional programming of various effector T cells (123). Naïve B cells are activated by encountering cognate antigens and migrate to the germinal center, where they undergo T cell-mediated co-stimulation and eventually specialize into antibody-secreting plasma cells. An estimated 30% of the compartment status of the genome has been changed in B- cell specification, most of which are in regions enriched for genes and TFs relevant to germinal center development (124). It appears that BCL6, which represses cell cycle checkpoint genes and is specifically upregulated in germinal center B cells, is coordinated with other genes in a way that facilitates proliferation by merging the 3D gene neighborhoods on chromosomes (125).

In summary, the activation of lymphocytes is followed by comprehensive, multistep 3D genome organization dynamics that enable cell proliferation and the formation of an inflammatory transcription program.

## 3D genome disorganization in autoimmune diseases

**Figure 3:**
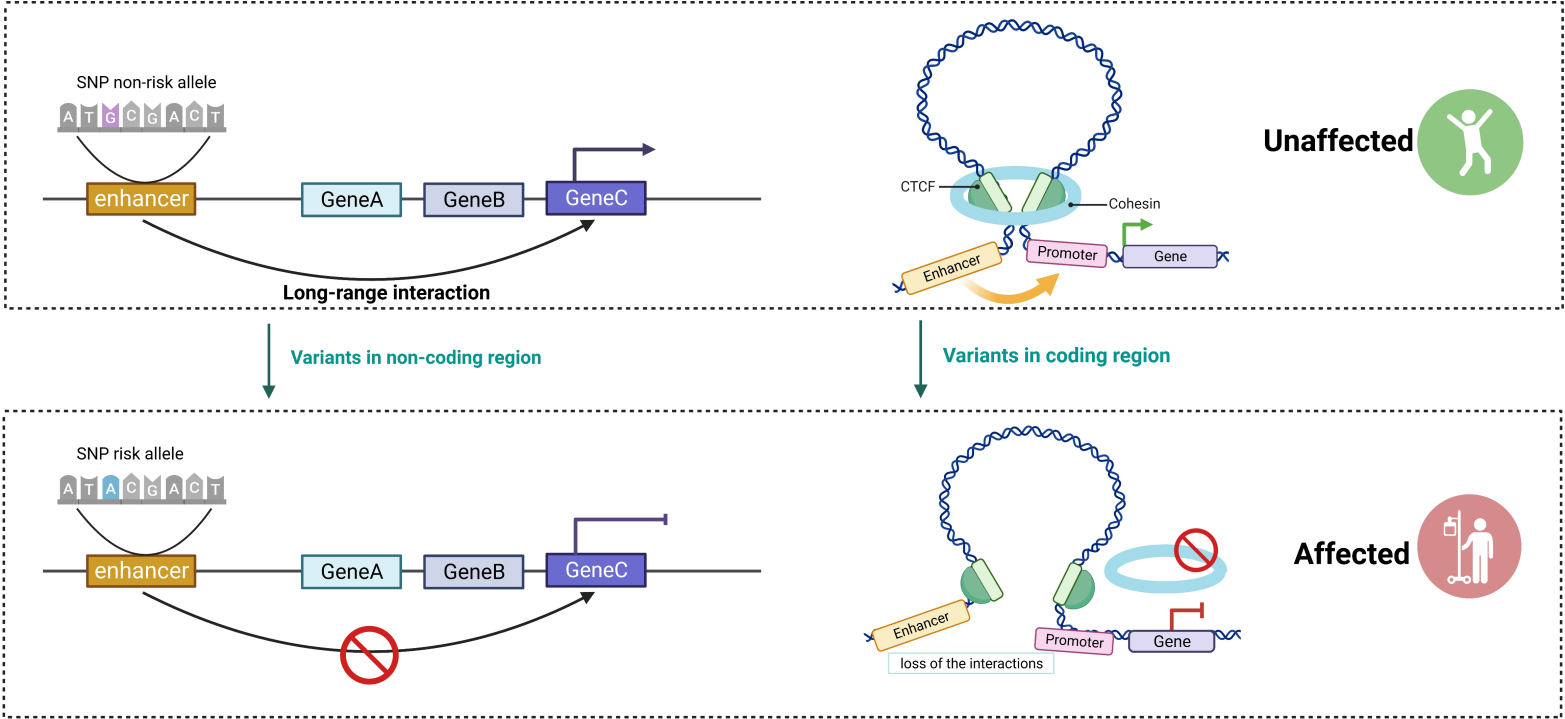
The critical role of 3D genome in the regulation of gene expression. Disruptions in gene expression caused by altered 3D architecture are frequently associated with immune disorders and can be classified into two scenarios. Altering non-coding sequences disrupts the binding of transcription factors (TFs) and impairs long-range interactions, resulting in reduced delivery of TFs to the promoter and hindering gene expression. Variants in the genes that encode proteins (such as TFs and cohesin complex) lead to changes in genome conformation (created with BioRender.com).

Immune cell dysfunction is responsible for a number of human morbidities, especially autoimmune diseases. When self or harmless antigens cause aberrant activation of lymphocytes, autoimmunity may be the result (126). Disruption of gene expression through altered 3D architecture is frequently associated with immune disorders and could be identified in two scenarios (101) (**Figure 3**). First, the alteration of non-coding sequences disrupts the binding of TFs or loop extrusion proteins, resulting in changes in 3D genome conformation and disrupting genomic conformation and transcriptional control. As GWAS variants are enriched in largely uncharacterized enhancer regions, it is essential to analyze their functional significance in the context of the 3D genome in order to understand how they influence disease pathology by reducing or enhancing interactions with the enhancer–promoter regulatory network (13). For instance, SNPs in non-coding regions interacting with tumor necrosis factor alpha-induced protein 3 (*TNFAIP3*) hindered TF binding and disrupted 3D chromatin folding in human CD4^+^ T cells (127). Second, variants in the genes that encode proteins (such as TFs) can also alter their function and alter the order of the 3D genome. For example, SATB1, a chromatin assembly protein and tissue-specific TF, is associated with T- cell development (128). Conditional depletion of SATB1 results in an autoimmune-like phenotype in mice because the obstructed promoter–enhancer loops in *Satb1* cKO T cells result in downregulated expression of genes encoding for master regulators (such as Bcl6, Ets2, and Cd8b) and T- cell receptor locus (129, 130).

CD4^+^ T cells are at the center of the adaptive immune system, and activation of T cells is a key etiological pathway involved in many autoimmune disorders (131). A total of 245 candidate disease genes for autoimmune diseases were identified based on promoter CHi-C data in activated CD4^+^ T cells and genetic associations for five autoimmune diseases. An example was IL2RA, in which allele-specific expression analyses were consistent with its interaction-mediated regulation (132). Functional genomic analysis not only helps translate GWAS results into biologically meaningful disease mechanisms but also can be used to validate or develop new therapeutic targets based on disease risk loci. Martin et al. exploited CHi-C to identify prospective therapeutic targets in rheumatoid arthritis (RA), juvenile idiopathic arthritis, and psoriatic arthritis in T-helper and B-cell lines. They have discovered that 48 out of 454 potential illness genes were pharmacological targets; however, only 11 were addressed by rheumatic disease medications, providing an approach for repurposing current treatments (133). Similarly, H3K27ac HiChIP was used to identify genes associated with chromatin interactions with disease-associated SNPs of some inflammatory skin diseases. Therefore, by identifying genes enriched for disease-relevant pathways, drugs could potentially be repurposed and developed to target these pathways (134).

In this part, we summarize recent research on how the 3D genome affects gene regulatory processes in autoimmune diseases.

### Systemic lupus erythematosus

Autoantibodies, abnormal T and B lymphocyte function, and immune complex development are features of SLE, an autoimmune disease that causes damage to multiple organs. Analysis of coordinated gene expression patterns in SLE patients and healthy controls revealed deregulation in the IFN pathway that was dependent on disease activity, indicating that genome organization and structure play a significant role in gene expression and disease activity (135). In our previous study, we analyzed the 3D chromosome structure of CD4^+^ T cells from SLE patients, finding that it differed considerably from that of healthy controls and was closely related to the severity of the illness (136). By using Hi-C, RNA-seq, ATAC-seq motif enrichment analysis, and histone modifications, we discovered SLE-associated loops with differentially expressed genes (DEGs) in CD4^+^ T cells correlated with disease activity. Over 90% of SLE-associated loops are based on enhancer–promoter contacts. Moreover, it was found that the promoter area of the *DDX60L* gene overlapped with two SLE-associated loops. *DDX60L* is an ATP-dependent helicase gene that participated in the innate immune IFN antiviral response. SLE patients with higher SLE disease activity index scores have a stronger frequency of interactions on *DDX60L*. We also found that the active histone marker and TF SPI1 were enriched at the two loop anchors. When ChIP-qPCR and 3C-qPCR were performed in SPI1 knockdown CD4^+^ T cells, silencing of SPI1 reduced the binding of SPI1 to the *DDX60L* promoter and decreased the association between the distal looping region and the *DDX60L* promoter. In addition, some SLE-associated SNPs, such as rs13385731, rs2732549, and rs2245214, were found to overlap with activating enhancers based on the enrichment of ChIP-seq data from ENCODE. For example, the enhancer region containing rs13385731 forms a long interaction loop, which may affect LTBP1 local chromatin conformation and gene expression (136). GWASs and fine-mapping identified a number of genetic loci linked to increased susceptibility to SLE in the chromosome 6q23 region, including rs148314165 and rs200820567, which were found in a potential enhancer region revealed by ENCODE that is approximately 42 kb downstream of the gene TNFAIP3 (84). The gene TNFAIP3 is a likely target of the discovered enhancer, as it is a major negative regulator of pro-inflammatory nuclear factor kappa B (NF-κB) signaling, which is associated with many autoimmune diseases (137). Moreover, the risk allele (A/A) in the enhancer dramatically disrupts NF-κB binding and inhibits long-range DNA looping of the enhancer to the TNFAIP3 promoter, effectively decreasing TNFAIP3 expression and increasing IL20RA and IFNGR1 expression through TALEN (transcription activation-like effector nuclease)-mediated genome editing, HiChIP, 3C-qPCR, and dual-luciferase reporter assays (85, 138). TNFAIP3 loss-of-function mutations result in elevated inflammatory responses, autoantibody production, and inflammatory arthritis in humanized mouse models (86, 139). Genome-wide investigation into the function of autoimmune risk polymorphisms is made possible by the combination of transcriptomic and epigenomic data with GWASs (67). A recent study found that the allele rs13239597-A is strongly associated with SLE and able to regulate IRF5 expression. This was performed using CRISPR/Cas9 and luciferase assays as well as Hi-C and CHi-C data across multiple blood cell lines (90). A cell type-specific epigenetic landscape of SLE SNPs in adult immune cells was examined using ENCODE and Roadmap Epigenomics data from CD4^+^ T cells and CD19^+^ B cells, together with ChIP-seq data from neutrophils (140). Super-enhancers (SEs) are a wide region of clustered enhancers with a high and widespread enrichment of TFs, H3K4me1, and H3K27ac, resulting in a high capacity to increase the gene expression program (141). Abnormal SE activity mediated by STAT3 has been discovered in memory B- cell subsets and is crucial for B- cell maturation and differentiation (87).

TFH cells are a type of cell that is necessary for the production of anti-nuclear antibodies, which are characteristic of SLE. Su et al. produced detailed and precise maps of the open chromatin–promoter connections in human TFH cells by applying promoter-focused Capture C and ATAC-seq analysis, providing a way to link non-coding open chromatin regions and SNPs to specific regions. The majority of SLE-associated variants do not interact with the closest gene but rather with distant promoters, many of which have been implicated in TFH and SLE, including *BCL6* and *CXCR5*. Additionally, genes whose role in TFH/SLE disease biology was previously unknown were discovered through the combination of GWASs and 3D genomics. For example, the kinases HIPK1 and MINK1 were essential for the production of IL-21, which was required for T cell-mediated help for B- cell antibody production in SLE patients (142).

In studies of monocytes, the genomic region containing rs2431697 was identified to be a cell-type-specific enhancer through the combination of genetic, epigenomic, and high-throughput sequencing technologies, such as meta-analysis GWASs, ChIP-seq for H3K4me1 and H3K27ac from Roadmap, and ATAC-seq data. Moreover, CRISPR gene editing techniques were used to edit these disease susceptibility loci in cell lines, primary immune cells, and humanized mice. The DNA sequence containing the non-risk allele (rs2431697 C) binds NF-κB with a greater affinity and is more accessible that the risk allele (T), leading to an increase in miR-146a production in monocytes through a special chromosomal 3D structure proved by 4C-seq. It has been determined how miR-146a controls the inflammatory response in SLE (143). Different alleles have influenced the binding ability of TFs and chromatin accessibility, which could regulate the expression of susceptibility genes of lupus (19). GWASs have demonstrated at least 16 genetic variants significantly associated with autoimmune disorders in the IRF8 locus, but it is difficult to identify the functional loci due to linkage disequilibrium (LD) (15). To address this issue, a high-throughput screen for the disease-related susceptibility loci through the CRISPR array has identified a genomic region located approximately 64 kb downstream of IRF8 (rs2280381) as an enhancer that specifically regulates IRF8 expression in monocytes. The spatial structure of the enhancer region and IRF8 promoter was examined using 4C. It is interesting to note that the enhancer RNA AC092723.1 is necessary for the development of this spatial structure because it recruits TET1 to the IRF8 promoter by changing methylation levels and chromatin state (20). Recently, an analysis of innate immunity was conducted among human primary monocytes from SLE patients based on a high-resolution 3D landscape by integrated datasets from Hi-C, RNA-seq, ATAC-seq, and ChIP-seq. High-resolution chromatin interaction maps of human primary monocyte samples show differences from those of the THP1 cell line. In addition, the 3D chromatin maps revealed a great deal of diversity in the HLA region among individuals (144).

### Rheumatoid arthritis

RA is an inflammatory autoimmune disease that damages bone and cartilage through the release of cytokines by activated T cells, B cells, and monocytes in the synovial membranes (145). Most of the research on disease-associated alleles at region 6q23 has been conducted in relation to the gene TNFAIP3. McGovern et al. identified causal genes and refined the possible functional SNPs at the locus 6q23 by searching for distal chromatin interactions with CHi-C, validating with 3C, and further proving with cell type- and genotype-specific eQTLs and ChIP. The result showed that the variant-associated DNA fragment interacts *via* a chromatin loop not only with TNFAIP3 but also with the more distant IL20RA instead of OLIG3 in B and T cells. Furthermore, there are more interactions between enhancer and IL20RA in patients with the risk G allele of the SNP rs6927172 compared to those with the non-risk allele (91). The JAK–STAT pathway is activated by IL20RA, which also includes IL-19, IL-20, and IL-24. Constitutive JAK–STAT activation stimulates the formation of cytokines and causes the mobilization of immune cells in an inflammatory environment, resulting in damage to the underlying cartilage due to chronic joint inflammation and proliferative synovitis (146). ATAC-seq, RNA-seq, Hi-C, and Chi-C analyses were performed in human CD4^+^ T cells stimulated with anti-CD3/anti-CD28 for 24 h. This showed that approximately 30% of the long-range chromatin junctions undergo significant changes, while compartments or TADs remain unchanged after 24 h of TCR activation. By collecting nuclear RNA-seq data, genes such as MYC and FOXO1 not previously implicated by GWASs in the RA pathophysiology have been found and also confirmed using CRISPR/Cas9 (147).

Although fibroblast-like synoviocytes (FLS) are crucial for the development of RA, there are few functional genetics databases for these cells. FLS samples from patients with RA were studied to characterize 3D chromatin interactions, DNA accessibility, and gene expression (148). Epigenetic and transcriptomic datasets of primary FLS samples were generated using ChIP-seq, ATAC-seq, RNA-seq, cap analysis gene expression sequencing (CAGE-seq), and chromatin conformation analysis (HiC and Chi-C). Up to 24% of RA heritability can be attributed to FLS, suggesting FLS’s role in influencing RA genetic risk independently. Moreover, the topological organization, chromatin state, and FLS samples from patients with RA were studied to characterize 3D chromatin interactions; the DNA LS genome was found to be significantly altered following TNF stimulation. RBPJ is a candidate causal and functional FLS gene based on epigenetic and functional analyses. Three SNPs (rs7441808, rs35944082, and rs874040) within the RBPJ region were selected to confirm enhancer activity by luciferase reporter assay and CRISPRi, which revealed that rs874040-containing enhancers regulate RBPJ expression (149).

### Multiple sclerosis

Multiple sclerosis (MS), which is characterized by persistent demyelination, oligodendrocyte mortality, and loss of axons and neurons, is an excellent illustration of an autoimmune disorder driven by abnormal activation of tissue-derived immune cells (150). Some chromosomal regions (such as 6q23 and 16p13) have been found to be associated with MS predisposition through GWASs (92, 151). The region rs11154801 and a region with the other three independent MS associations (rs17066096, rs7769192, and rs67297943) appeared to form the majority of chromosomal interactions in 6q23. The SNP rs11154801 within the introns of AHI1 interacts with the promoter region and correlates with expression, supporting the gene candidacy of AHI. The CHi-C results indicated that the other interaction was linked to multiple immune-related genes, such as IL20RA, *IL22RA2*, *IFNGR1*, and *TNFAIP3*, suggesting that these variants could play a role in regulating numerous immune pathways (92). SNPs at or close to CLEC16A at chromosome 16p13.13 including rs12927355, rs4780346, rs12708716, and rs8062322 serve as expression quantitative trait loci for CIITA, SOCS1, and DEXI in peripheral T cells. In samples homozygous for the CLEC16A rs12927355 risk allele, SOCS1 and CLEC16A were expressed at a higher level on CD4^+^ T cells (152). The functions of CIITA and SOCS1 in immune cells have been established. CIITA encodes the MHC class II transactivator, which is an important genetic factor that determines susceptibility to autoimmune diseases (153). SOCS1 plays a role in inhibiting type I interferon signaling and regulating inflammation and immune cell homeostasis (154). Nevertheless, *DEXI* is a gene considered to respond to dexamethasone, a pro-inflammatory drug, and DEXI probably regulates inflammation (151).

### Type 1 diabetes

Even though diabetes is regarded as a metabolic disorder, exogenous insulin dependency is typically caused by the autoimmune destruction of insulin-producing cells of the pancreas in type 1 diabetes (T1D). GWASs have found over 60 loci associated with T1D across the human genome (155). To identify genes regulated by T1D-associated genetic variants, Nyaga et al. performed a combined spatial and functional eQTL analysis using the CoDeS3D algorithm. A total of 246 spatially regulated genes, including HLA-DRB1, LAT, CTLA4, and NOTCH1, which exhibit tissue-specific effects in multiple tissues, were found based on the Genotype-Tissue Expression database. Moreover, T1D-associated variants are interconnected through networks that comprise immunological regulatory mechanisms, including immune-cell activation, cytokine, and programmed cell death protein-1 (PD-1) (156). Genetic variation in an intron of CLEC16A acts at a distance to regulate the promoter region of DEXI, and decreased DEXI expression is associated with an increased risk for T1D (157). The non-obese diabetic (NOD) mouse strain offers an excellent model for understanding the complex processes associated with autoimmune diseases. In T cells from T1D mice, H3K27ac and Smc1 HiChIP revealed hyperconnected clusters in T1D-associated regions, including genes associated with autoimmunity, including BCL11B and ETS1, but these clusters were not found in WT mice. However, when NOD mice were backcrossed with WT mice, decreased 3D chromatin interaction was identified in disease-related regions, suggesting that genomic variants deregulating enhancer function were responsible. The 3DFAACTS-SNP workflow identified 47 new candidate genes associated with variants in 12 T1D risk loci related to loss of immune tolerance in regulatory T cells. For example, SNP rs614120 located in the first intron of the BACH2 was predicted to inhibit the binding of FOXA2 (a member of the Forkhead Transcription factor family). It is noteworthy, however, that rs614120 may interfere with the binding of other members of the forkhead family, such as FOXP3, to similar DNA sequences due to the lack of expression of FOXA2 in T cells. The 3DFAACTS-SNP procedure also reveals that this rs614120-containing enhancer region interacts with the BACH2 promoter to create a distal promoter–enhancer interaction. This suggests that rs614120 can interfere with the binding of FOXP3 to the enhancer and lead to dysregulated BACH2 production (93).

### Other diseases

CD4^+^ T cells are one of the principal drivers of pathogenesis in systemic sclerosis (SSc), a highly complex autoimmune disease regulated by the interaction of hereditary and epigenetic factors (158). DNA methylation surrounding HLA gene clusters and genes enriched in inflammatory pathways differed between SSc patients and healthy controls in CD4^+^ T cells. A study linking genetic risk to epigenome and transcriptome dysregulations has identified four significant risk genes for SSc, TNIP1 (rs3792783), GSDMB (rs9303277), IL12RB1 (rs2305743), and CSK (rs1378942), which potentially interact with DMP-DEG (differentially methylated CpG position–differentially expressed gene) pairs cg17239269–ANXA6, cg19458020–CCR7, cg10808810–JUND, and cg11062629–ULK3, respectively (159). Moreover, CTCF binding was enriched in DMPs in CD4^+^ cells from SSc patients, which may be a result of abnormal overexpression of the CTCF gene (159). CTCF recruitment, previously characterized as being DNA methylation-dependent, would be deregulated by aberrant DNA methylation and also participate in long-distance contacts that affect aberrant gene regulation (160). Takayasu arteritis is linked to genetic susceptibility loci primarily in non-coding regions, similar to other autoimmune diseases. To identify chromatin looping in the IL-6 locus, DNA-affinity pulldown assay was applied, followed by mass spectrometry and Western blotting, luciferase reporter assays, and 3C. A change in the intron domain of IL-6 (rs2069837) could disrupt a chromatin loop and recruit the repressive HDAC–MEF2 complex, which inhibits the anti-inflammatory monocyte gene GPNMB, mediating increased susceptibility to Takayasu arteritis and promoting persistent infections and cancer (94). Inflammatory bowel diseases (IBDs), including ulcerative colitis (UC) and Crohn’s disease (CD), are chronic inflammatory disorders that affect the colonic mucosa and gastrointestinal tract, respectively. Genetics participates in the pathogenesis of IBD, as demonstrated by twin, family, and population-based studies (161). Based on H3K27ac analysis, 92 of 163 IBD risk loci are localized to DNA regulatory elements (DREs), particularly enhancer elements in the intestinal epithelium and immune cells (162). Following 4C-seq in monocytes, lymphocytes, and intestinal epithelial cells, 902 new IBD candidate genes interacting with 92 DRE were discovered. In monocytes and lymphocytes, these genes were enriched for signaling in immune pathways such as JAK–STAT and interferon. I ntestinal epithelial cells, however, were enriched for signaling in IL-10, RHO, and RAC (163). In CD patients, SNP rs6651252 is associated with Wnt-responsive DNA enhancer elements, and its disease-associated allele boosts TF TCF7L2 binding. By employing CRISPR/Cas9 and epigenetic modulation, the rs6651252 enhancer was found to regulate the expression of the c-MYC proto-oncogene (95). By performing systematic annotation, prediction, and prioritization of the known pleiotropic variants associated with autoimmune diseases from 12 genome-wide studies, rs4728142 was identified as a top causal regulatory variant that was located upstream of the pro-inflammatory gene *IRF5*. ZBTB3, a putative structural regulator, mediates the formation of site-specific chromatin loops in the chromosomal rs4728142 locus. The presence of the rs4728142-A risk allele facilitated the tight binding of ZBTB3 and regulated the upregulation of IRF5-induced genes related to autoimmunity. This process leads to the polarization of M1 macrophages, contributing to the occurrence and development of autoimmune diseases by improving the inflammatory and immune response (96) (**Table 2**).

**Table 2:** The regulatory effects of SNPs in non-coding regions in the autoimmune diseases.

Disease	*Target Gene*	Variant ID	Ref/Alt	Position	Cell specificity	Phenotype changes	Main research techniques	Reference
**SLE**	***TNFAIP3***	rs148314165	TT/A^*^	chr6:138230037	EBV-transformed B cell lines	TT>A variants has an inability to effectively deliver NF-κB to the *TNFAIP3* promoter through long-range DNA looping.	ChIP-PCR, 3C-qPCR, EMSA, MS, luciferase assay	(84)
		rs200820567		chr6:138230039				
		rs10499197	T/G	chr6:138132515	EBV-transformed B cell lines;	CEBPB and RelA binding motifs increase risk allele-specific expression of IL20RA and IFNGR1 with long-range looping	ChIP-qPCR, 3C-qPCR, HiChIP EMSA, Dual-Luciferase reporter assays	(85)
		rs58905141	A/G	chr6:138132122		risk allele significantly reduced nuclear protein complex binding		
		rs9494868	T/G	chr6:138132444		risk allele-specific increase in nuclear factor binding and enhancer activation		
	***TNIP1***	rs10057690	T/C	chr5:150445214	EBV-transformed B cell lines	reduced enhancer activity	ChIP-qPCR, HiChIP, EMSA, luciferase reporter assays, DAPA	(86)
		rs13180950	T/C	chr5:150452552		increased enhancer activity		
		rs10036748	C/T	chr5:150458145		reduced binding affinities of transcriptional repressors, Bhlhe40/DEC-1 and CREB-1, in EBV B cells, resulting in a gain of enhancer activity		
	***STAT3***	rs1047643	T/C	chr8:11660361	GM11997 B lymphoblastic	associated with STAT3-mediated super enhancer activity and B cell deregulation	ATAC-seq, RNA-seq, eQTL, ChIP-qPCR, Hi-C	(87)
	***miR-146a***	rs57095329	A/G	chr5:159894846	Jurkat T cells	influencing miR-146a expression by altering its binding affinity for Ets-1	Luciferase reporter assays, EMSA, Streptavidin–agarose pulldown and western blotting	(88)
		rs2431697	T/C	chr5:159879977	U-937; human CD14+ monocytes	modifying NF-κB binding and chromatin state to downregulate the expression of miR-146a and activation of the type I interferon pathway	eQTLs, ChIP, FAIRE-qPCR, 4C-seq, RNA-seq, ATAC-seq, CRISPRa, CRISPRi, CRISPR-Cas9, Cas9 RNP, allele-specific qPCR, DAPA, LC-MS	(19)
	***IRF8***	rs2280381	C/T	chr16:86018632	U-937; human CD14+ monocytes	mediating IRF8 expression through enhancer RNA AC092723.1, which recruits TET1 to the IRF8 promoter by affecting methylation levels	4C-seq,ChIP-qPCR, FAIRE-qPCR, Allele-specific qPCR, ChIRP- qPCR, siRNAs, RIP-qPCR, CRISPRa/i, CRISPR-Cas9, DAPA, LC-MS, luciferase reporter assay	(20)
	***IRF5***	rs4728142	G/A	chr7:128573966	SLE patients' monocytes.	inhibiting ZBTB3 binding, chromatin status, and IRF5 expression	AS-ChIP-qPCR, FAIRE-qPCR, eQTL, CRISPRa, CRISPRi, CRISPR-Cas9	(89)
**SLE/ SSc**	***IRF5***	rs13239597	C/A	chr7:128695982	EBV-transformed B cell lines (Raji)	binding the TF EVI1 and increasing the enhancer activity to regulate IRF5 expression	**eQTL, 3C, Motif analysis, CRISPR-Cas9, shRNA knockdown, ChIP**	(90)
**RA**	***TNFAIP3***	rs6927172	C/G	chr6:138002174	Jurkat T cells, human CD4+ T cells	increasing expression of IL20RA through enhacing regulatory activity and binding of the NF-κB.	**Capture Hi-C, 3C, eQTLs, ChIP,**	(91)
**MS**	***AHI1***	rs11154801	C/A	chr6:135739354	B-lymphoblastoid cell lines (LCL), Jurkat T-lymphoblast cells	increasing expression of AHI1 by interacting with its promoter	Capture Hi-C, eQTLs	(92)
	***IFNGR1***	rs17066096	A/G	chr6:137452907		interacting with each other and with immune-related genes such as IL20RA, IL22RA2, IFNGR1 and TNFAIP3		
	***IL20RA***	rs7769192	G/A	chr6:137962654				
	***TNFAIP3***	rs67297943	T/C	chr6:138244815				
**T1D**	***BACH2***	rs614120	C/G	chr6:90995979	Human Treg cells	dysregulating the expression of BACH2 by interfering with *FOXP3* binding to the enhancer	**ChIP-seq, ATAC-seq, Hi-C, eQTL,**	(93)
**Takayasu arteritis**	***IL6***	rs2069837	A/G	chr7:22768026	THP-1 cell line, human fibroblast-like macrophages	repressing the expression of GPNMB by recruiting MEF2–HDAC complex	**3C, DAPA-MS, luciferase reporter assays**	(94)
**CD**	***MYC***	rs6651252	T/C	chr8:129567180	HCT116 and DLD-1 cell lines	facilitating stronger TCF7L2 binding and increasing MYC gene expression	**ChIP, luciferase reporter assays, CRISPR/Cas9, DAPA**	(95)
	***IRF5***	rs4728142	G/A	chr7:128573966	Human peripheral blood monocyte-derived SC cell line	increasing expression of IRF5 and leading to M1 macrophage polarization	**ATAC-seq, ChIP-seq, luciferase reporter assay, 3C, 4C, RNA-seq, EMSA, CRISPRa, CRISPRi**	(96)

SLE, Systemic lupus erythematosus; RA, Rheumatoid arthritis; MS , Multiple sclerosis; T1D, Type 1 diabetes; CD, Crohn's disease; TNFAIP3, tumor necrosis factor alpha inducible protein 3; NF-κ B, nuclear factor kappa B; EMSA, electrophoretic mobility shift assays, MS, mass spectrometry, reporter assays, ChIP-PCR:chromatin immunoprecipitation-PCR; 3C, chromosome conformation capture; Hi-C, high-throughput chromosome conformation capture; HiChIP:Hi-C followed by chromatin immunoprecipitation; eQTL, expression quantitative trait loci; DAPA, DNA-affinity pulldown assay; LC-MS:Liquid Chromotography with Mass Spectrometry; RIP, RNA immunoprecipitation; ChIRP:Chromatin isolation by RNA purification;

*variants rs148314165 (-T) and rs200820567 (T>A), referred to as TT>A.

## Conclusion and future perspective

As previously mentioned, the application of 3D genomics has significantly advanced our knowledge of the genetics of lymphocyte development and the etiology of autoimmune diseases. The 3D genome can help to determine the functional effect of disease genetics on the associated target genes, involving the gene expression and regulatory networks or pathways, which provides a great grasp of the mechanisms of spatial conformational changes in chromatin, transcriptional regulation, and the generation of biological features. Our previous research identified the affecting gene regulatory elements, such as active enhancers and promoters within the genome loops, revealing the new mechanisms of SLE from the 3D genome landscape (136).

GWASs are based on the assumption that common diseases are often influenced by a large number of genetic variants. After analyzing information from Hi-C sequencing, histone markers, and chromatin accessibility, some risk-associated SNPs were found to be located in the promoter or enhancer region. Risk variants can affect gene expression by interacting with distal genomic regions through 3D chromatin folding. These interactions can bring the regulatory elements closer to the target gene and increase or decrease its expression levels, depending on the nature of the interaction and the specific regulatory elements involved. However, it is especially challenging to demonstrate the functions of enhancers through experiments. Through targeting genomic deletions and mutations, CRISPR/Cas9 and CRISPR-based approaches, such as CRISPRi and CRISPRa, were used to identify the function of potential regulatory regions coupled with transcriptional quantification or RNA-seq. The integration of GWASs and omics data allows the elucidation of the function of individual risk variants and the identification of disease and tissue specificity. This helps to elucidate the detailed pathogenesis process of autoimmune diseases and thus leads to precise diagnosis and treatment.

Although 3C and its derivatives are unquestionably powerful tools for structural and functional genomics research, those technologies still face challenges on several fronts, such as difficulties in detecting weak interactions between chromatin, long experimental cycles that do not allow for a rapid and realistic response to interactions and a low ratio of signal to noise. Moreover, current visualization tools are incapable of integrating genomic information across the interaction intensity, spatial conformation, and gene synergy, as 3D genome data indicate the spatial composition and functional interconnections in multiple dimensions. The spatial conformation of the genome in the nucleus varies with time in order to sustain proper cell growth and development. As the 4D nucleosome project proceeds, it will be feasible to map the spatial and temporal organization and dynamics of the genomic structure of humans (164). There are still some challenges in the clinical application. For example, how to manipulate the structure of the chromatin or alter the genetic loci to investigate their impact on the disease process is still a problem. It is also necessary to create novel treatments for illnesses based on genomic information.

## Author contributions

YQ, QL, and MZ made substantial contributions to the study’s conception and design. All authors drafted the article or revised it critically for important intellectual content. All authors gave their final approval of the version to be submitted and any revised versions.
